# Ethnic differences in prevalence and risk factors for hypertension in the Suriname Health Study: a cross sectional population study

**DOI:** 10.1186/s12963-016-0102-4

**Published:** 2016-09-17

**Authors:** Ingrid S. K. Krishnadath, Vincent W. V. Jaddoe, Lenny M. Nahar-van Venrooij, Jerry R. Toelsie

**Affiliations:** 1 Department of Public Health, Faculty of Medical Sciences, Anton de Kom University of Suriname, Paramaribo, Suriname; 2 Department of Physiology, Faculty of Medical Sciences, Anton de Kom University of Suriname, Paramaribo, Suriname; 3 Department of Epidemiology, Erasmus MC, University Medical Center, Rotterdam, The Netherlands; 4 Department of Pediatrics, Erasmus MC, University Medical Center, Rotterdam, The Netherlands

**Keywords:** Amerindian, Ethnicity, Hypertension, Risk factors, Suriname

## Abstract

**Background:**

Limited information is available about the prevalence, ethnic disparities, and risk factors of hypertension within developing countries. We used data from a nationwide study on non-communicable disease (NCD) risk factors to estimate, explore, and compare the prevalence of hypertension overall and in subgroups of risk factors among different ethnic groups in Suriname.

**Method:**

The Suriname Health Study used the World Health Organization Steps design to select respondents with a stratified multistage cluster sample of households. The overall and ethnic specific prevalences of hypertension were calculated in general and in subgroups of sex, age, marital status, educational level, income status, employment, smoking status, residence, physical activity, body mass index (BMI), and waist circumference (WC). Differences in the prevalence between ethnic subgroups were assessed using the Chi-square test. We used several adjustment models to explore whether the observed ethnic differences were explained by biological, demographic, lifestyle, or anthropometric risk factors.

**Results:**

The prevalence of hypertension was 26.2 % (95 % confidence interval 25.1 %-27.4 %). Men had higher mean values for systolic and diastolic blood pressure compared to women. Blood pressure increased with age. The prevalence was highest for Creole, Hindustani, and Javanese and lowest for Amerindians, Mixed, and Maroons. Differences between ethnic groups were measured in the prevalence of hypertension in subcategories of sex, marital status, education, income, smoking, physical activity, and BMI. The major difference in association of ethnic groups with hypertension was between Hindustani and Amerindians.

**Conclusion:**

The prevalence of hypertension in Suriname was in the range of developing countries. The highest prevalence was found in Creoles, Hindustani, and Javanese. Differences in the prevalence of hypertension were observed between ethnic subgroups with biological, demographic, lifestyle, and anthropometric risk factors. These findings emphasize the need for ethnic-specific research and prevention and intervention programs.

## Background

Hypertension is the fourth-largest contributor to premature death in industrialized countries and the seventh in developing countries [1–3]. The increasing prevalence of hypertension in developing countries could be the result of factors like urbanization, population aging, unhealthy dietary habits, and social stress [4]. In several industrialized countries, ethnic differences in the prevalence of hypertension and its risk factors have been described extensively [5–17]. In contrast, in developing countries less research has been conducted. Studies reported higher prevalence of hypertension among adults from African descent followed by those of Asian or Hispanic descent, as compared to Caucasians [7–9, 18–20].

The Republic of Suriname, located on the northeast of South America, is an upper-middle income country with a multi-ethnic and multicultural population, with inhabitants of mainly Indian, African, and Indonesian descent. In this country, cardiovascular disease has been the main cause of mortality for decades in each ethnic group [21]. However, information about the prevalence and risk factors of hypertension among these different groups is limited. So far, a study from 2001, limited to three coastal districts, reported a hypertension prevalence of 33 % in adults between the ages of 18-55 years [22]. Of all participants, 70 % were physically inactive, 30 % smoked, 20 % were obese, and 15 % had high total cholesterol levels. In adults, the highest prevalence of hypertension has been observed in Creoles [22]. In adolescents, hypertension was measured more frequently in Hindustani and Javanese [23].

We used data from a nationwide study on non-communicable disease (NCD) risk factors [24], to estimate, explore, and compare the prevalence of hypertension overall and in subgroups of biological, demographic, lifestyle, and anthropometric risk factors among different ethnic groups in Suriname.

## Methods

### Design

We used data from the Suriname Health Study, the first nationwide study on NCD risk factors [24], which was designed according to World Health Organization (WHO) Steps guidelines [25]. The Ethics Committee of the Ministry of Health in Suriname (Commissie mensgebonden wetenschappelijk onderzoek (ref: VG 004-2013)) approved this research. Suriname has approximately 550,000 inhabitants, categorized into 15.7 % Creole (descendants of African plantation slaves), 27.4 % Hindustani (people of Indian heritage), 13.7 % Javanese (descendants from Indonesians), 21.7 % Maroon (descendants of African refugees who escaped slavery and formed independent settlements in the hinterland), 13.4 % mixed, 7.6 % others, including Amerindians (original inhabitants), and 0.6 % unknown [26]. Because of financial restraints and the extended survey area in Suriname we used a stratified multistage cluster sample of households to select respondents between March and September 2013 for this study [24]. The strata were based on the geographic location of the sampling units in the various districts. The Primary Sampling Unit (PSU) of the sampling frame consists of the 10 districts of Suriname. The Secondary Sampling Units (SSU) consisted of 101 randomly selected enumeration areas (EAs) in nine districts and four randomly selected village areas in a remote tenth district, Sipaliwini. The SSU was divided into 343 clusters, which were selected randomly within the enumeration areas. Except for the 16 clusters in district Sipaliwini, each cluster contained 25 households. The clusters in Sipaliwini contained 40 households, due to the large cost of transportation to the isolated villages in the tropical rainforest. In the selected households, the respondent was identified using the Kish method based on gender and age [27]. In total, 7493 individuals between the age of 15 and 65 years were invited to participate in the study. The response rate was 76.8 %, resulting in 5748 participants. The percentage of missing data was relatively small (1.1 %) for most variables except for income status (30.2 %) [24].

### Main outcome

We measured blood pressure three times with the Omron HEM-780 blood-pressure monitor.

Before measurements, respondents were seated (legs uncrossed) to rest for at least 15 min. Measurements were repeated at a time interval of three minutes. The mean of the last two measurements was used to calculate the blood pressure of the participant [28]. Hypertension was defined as a systolic blood pressure ≥140 mm Hg, diastolic pressure ≥90 mm Hg, or current treatment with antihypertensive medication [29].

Hypertensive respondents were considered aware of their condition when previously diagnosed and using antihypertensive medication. Additionally, they were considered to have their hypertensive condition under control when they used antihypertensive medication and had measurements of <140 mm Hg for systolic blood pressure and <90 mm Hg for diastolic blood pressure.

### Risk factors

We used interviews and hands-on measurements to collect information. Trained interviewers used an adapted WHO steps questionnaire to collect demographic and lifestyle information. Participants were categorized into a specific ethnic group if at least three of four grandparents were of this ethnicity. Anybody else was considered to be of mixed ethnicity. Next to ethnicity, we considered biological factors like sex and age; demographic factors like marital status, educational level, income status, employment, and residence (stratified into urban, rural coastal, and rural interior areas); lifestyle factors like cigarette smoking and physical activity (in Metabolic Equivalent of Task (MET) minutes); and anthropometric factors like body mass index (BMI) and waist circumference (WC) as risk factors for hypertension. The Global Physical Activity Questionnaire (GPAQ) was used to measure physical activity. All measurements were carried out as described in Part 3 “Training and Practical guides” of the WHO steps manual with the recommended equipment [28]. Height was measured with the Seca 213 stand-alone stadiometer, and WC was determined with the Seca 201 measuring tapes. The respondents were weighed with the Tanita HS302 solar scale. BMI was classified in the categories; <23, 23-25, 25-27.5, 27.5-30 and 30+, taking into account the WHO ethnic-specific cutoff points for overweight and obesity. According to WHO, a WC of ≥88 cm for women and ≥102 cm for men is associated with substantially increased metabolic risk [30]. We applied the values above this WC cut off point to classify the waist as large. Household income was classified as the income status quintile from the Ministry of Internal Affairs of Suriname in Surinamese dollars, SRD (1USD = 3.35 SRD). Because of the small amount of respondents in the fourth and fifth quintile, these two were combined in the analysis. The residential addresses were stratified to urban, rural coastal areas, and the rural interior [31]. Physical activity was classified according to WHO recommendations in groups <600 MET and ≥600 MET.

### Statistical analysis

First, we calculated the overall and ethnic-specific prevalence of hypertension and the main risk factors. Differences in the prevalence between ethnic subgroups were assessed using the Chi-square test. The collected data were subjected to a weighting procedure so inferences could be made to the whole population. The weights used for analysis were calculated to adjust for probability of selection, non-response, and differences between the sample population and target population. The adjustment weight for sample correction was calculated per sex, 10-year age group, and, where applicable, ethnicity [24]. Second, we used several adjustment models to explore whether the observed differences in association were influenced by biological, demographic, lifestyle, or anthropometric risk factors. We tested potential interactions between Hindustani (the group with the highest prevalence of hypertension from the two largest groups) and other ethnic groups, adjusted for sex and age in relation to modifiable cardiovascular risk factors. All models were evaluated with the Hosmer and Lemeshow goodness to fit test. We used the statistical software Epi Info 3.2 and the Statistical Packages for Social Sciences (SPSS 21.0) for analyses.

## Results

### Subject characteristics

Table 1 presents the subjects’ characteristics of the study population overall and per ethnic subgroup. Amerindians and Maroons had the lowest percentages of male participants and smokers and the highest percentages of subjects with low education, low income, and living in rural interior areas. Also, Amerindians had the highest BMI and WC but the lowest mean systolic blood pressure. Compared to the other ethnic groups, Creoles had the highest percentages of male participants and high education and income groups. Creoles had the lowest percentage of subjects living with a partner and the highest percentages of subjects who were employed or met the required level of physical activity. Also, they had the highest mean systolic blood pressure. Creoles, Hindustani, and Javanese exhibited low levels of living in rural interior areas. Hindustani subjects had the highest mean diastolic blood pressure. Javanese had the highest median age and the highest percentages of living with a partner and smoking. Maroons had the lowest median and mean values for BMI and WC.

**Table 1:** Subject characteristics, overall and by ethnic subgroup (*N* = 5641)

Characteristics	Overall	Amerindian	Creole	Hindustani	Javanese	Maroon	Mixed
	*N* = 5641	*N* = 427	*N* = 677	*N* = 1315	*N* = 923	*N* = 1377	*N* = 817
Men % (95 % CI)	49.7(48.4-51)	37.6(31.7-44)	54.4(50.6-58.2)	52.8(50.4-55.2)	51.2(47.7-54.6)	43.2(40.3-46.2)	46.3(43.1-49.6)
Age in years, median (95 % range)	35.0(15.0-62.0)	35.0(16.0-62.0)	36.0(16-62)	37.0(16.0-62.0)	40.0(15.0-62.2)	30.0(15.0-61.0)	32.0(15.0-62.0))
Education % (95 % CI)							
Low	53(51.7-54.3)	78.6(73-83.5)	37.5(33.8-41.4)	54.3(51.8-56.7)	53.3(49.8-56.8)	77.3(74.7-79.8)	34.8(31.7-38.0)
Middle	27.9(26.7-29.1)	17.8(13.2-23.1)	34.8(31.1-38.6)	27.9(25.7-30.1)	32(28.8-35.4)	14.1(12.1-16.4)	35.4(32.3-38.6)
High	19.1(18.1-20.2)	3.6(1.7-6.8)	27.7(24.4-31.4)	17.9(16.1-19.8)	14.7(12.4-17.4)	8.5(7-10.4)	29.8(26.9-32.9)
Income % (95 % CI)							
< SRD 800/month	33.6(32-35.2)	54.7(46-63)	23.0(19.0-27.6)	30.9(28.0-34.0)	23.9(20.3-27.9)	58.3(54.4-62)	23.4(19.9-27.3)
SRD 800-1499/month	33.9(32.3-35.5)	26.4(19.3-34.4)	39.9(35.0-45.0)	37.6(34.6-40.8)	37.8(33.6-42.2)	27.6(24.3-31.2)	28.3(24.6-32.4)
SRD 1500-2199/month	15(13.8-16.3)	7.2(3.5-12.7)	16.2(12.8-20.4)	17.2(14.9-19.7)	19.4(16.1-23.2)	7.5(5.6-9.8)	16.8(13.7-20.3)
SRD 2200/month	17.5(16.2-18.8)	11.7(6.7-17.9)	20.9(17.0-25.3)	14.3(12.2-16.7)	18.9(15.6-22.6)	6.7(5.0-8.9)	31.5(27.6-35.7)
Area % (95 % CI)							
Urban coastal	75.5(74.3-76.6)	31.2(25.4-37.2)	85.8(82.9-88.3)	83.9(82-85.6)	70.7(67.4-56.8)	52.3(49.2-55.3)	85.4(83-38)
Rural coastal	16.1(15.1-17)	32(26.1-38)	14.1(11.7-17)	16.1(14.4-18)	29.3(26.2-35.4)	6.9(5.5-8.7)	14.3(12.2-38.6)
Rural interior	8.5(7.8-9.2)	36.9(30.9-43.2)	0(0-0.7)	0(0-0.3)	0(0-17.4)	40.8(37.9-43.8)	0.3(0.1-32.9)
Living with partner % (95 % CI)	51.7(50.4-53)	66(59.7-71.7)	32.3(28.8-36)	60.4(58-62.7)	70.7(67.4-73.8)	40.1(37.2-43.1)	44(40.8-47.2)
Working or studying % (95 % CI)	69(67.8-70.2)	44.3(38.2-50.8)	78.7(75.4-81.6)	67.8(65.5-70)	71.9(68.7-74.9)	58.7(55.7-61.6)	77.2(74.3-79.8)
Smoking % (95 % CI)	14.7(13.8-15.6)	5.3(2.8-8.7)	19.1(16.2-22.3)	16.2(14.5-18.1)	20.2(17.5-23.1)	6.5(5.1-8.2)	14(11.9-16.4)
Recommended physical activity % (95 % CI)	64.3(62.9-65.6)	66.3(59.1-73.1)	69.6(65.7-73.3)	65.4(63-67.8)	60(56.3-63.5)	59.2(55.9-62.5)	65.6(62.2-68.8)
Body mass index, kg/m^2^, median (95 % range)	25.7(25.6-25.9)	26.7(18.4-40.7)	25.5(17.6-41.0)	26.3(17.1-39.1)	26.1(17.8-40.1)	24.9(17.5-41.3)	25.8(17.2-39.8)
Waist circumference, cm, mean (SD)	87.3(15.6)	90.0(14.4)	88.2(16.3)	89.4(15.7)	86.0(14.9)	84.6(15.0)	86.4(16.0)
Systolic blood pressure, mmHg, mean (SD)	119.0(19.3)	117.3(18.6)	122.4(19.7)	119.1(19.3)	119.3(19.5)	118.4(18.9)	117.5(19.5)
Diastolic blood pressure, mmHg, mean (SD)	78.6(12.7)	76.3(11.4)	79.4(13.2)	79.6(12.3)	79.4(13.0)	77.3(13.2)	77.4(12.2)

The values are estimated means (standard deviation, SD), medians (range) or proportions (confidence interval, CI) and are based on weighted data. The sample weights included population adjustment weights for sex and age. For analysis on the overall population, additional adjustment weights for ethnicity were included. The sample of the overall population included the presented ethnic subgroups (*n* = 5536) plus other unspecified ethnicities (*n* = 105)

Table 2 shows that the mean systolic and diastolic blood pressure is higher in men and increases with age, whereas the mean BMI was higher in women and increases with age.

**Table 2:** Mean systolic and diastolic blood pressure and mean BMI by age and sex

Age group	Systolic blood pressure		Diastolic blood pressure		BMI	
	Mean ± SD (SE)		Mean ± SD (SE)		Mean ± SD (SE)	
	Male	Female	Male	Female	Male	Female
	(*n* = 2102)	(*n* = 3524)	(*n* = 2102)	(*n* = 3522)	(*n* = 2052)	(*n* = 3302)
15-24 years	115.1 ± 12.6 (0.5)	102.6 ± 11.1 (0.4)	71.5 ± 9.8 (0.4)	69.5 ± 8.4 (0.3)	23.0 ± 4.8 (0.2)	24.2 ± 5.2 (0.2)
25-34 years	120.3 ± 13.0 (0.5)	108.5 ± 14.4 (0.5)	78.3 ± 10.6 (0.4)	75.2 ± 11.8 (0.4)	25.7 ± 5.2 (0.2)	27.9 ± 5.9 (0.2)
35-44 years	123.5 ± 15.6 (0.6)	117.4 ± 18.4 (0.8)	82.3 ± 11.5 (0.5)	81.2 ± 12.9 (0.5)	26.5 ± 5.2 (0.2)	29.1 ± 6.1 (0.3)
45-54 years	128.5 ± 19.0 (0.8)	126.3 ± 20.6 (0.9)	84.3 ± 12.5 (0.5)	83.6 ± 12.2 (0.5)	26.1 ± 5.1 (0.2)	29.2 ± 5.8 (0.3)
55-64 years	137.9 ± 23.6 (1.4)	134.6 ± 22.0 (1.2)	86.6 ± 13.4 (0.8)	84.3 ± 12.3 (0.7)	26.2 ± 5.2 (0.3)	29.7 ± 6.3 (0.3)
Total pop.	123.1 ± 17.5 (0.3)	115.2 ± 20.1 (0.4)	79.4 ± 12.5 (0.2)	77.5 ± 12.8 (0.2)	25.3 ± 5.2 (0.1)	27.7 ± 6.2 (0.1)

The values are estimated means ± the standard deviation (SD) with the standard error (SE) and are based on weighted data. Total pop. = total population

Table 3 shows that a higher percentage of men smoke and comply with the recommended levels of physical activity. In both sexes, smoking increased and physical activity decreased with age.

**Table 3:** Prevalence of smoking and weekly required physical activity by age and sex

Age group	Smoking		Recommended physical activity	
	%(CI 95 %)		% (CI 95 %)	
	Male	Female	Male	Female
	(*n* = 2093)	(*n* = 3533)	(*n* = 1784)	(*n* = 3070)
15-24 years	8.8(6.9-11.2)	0.6(0.2-1.5)	82.2(78.9-85.2)	61.9(58.0-65.7)
25-34 years	23.1(20.0-26.6)	3.8(2.6-5.6)	75.5(71.6-79.0)	55.8(51.7-59.9)
35-44 years	28.9(25.3-328)	5.6(3.9-7.9)	68.7(64.4-72.8)	57.0(52.6-61.4)
45-54 years	35.9(31.8-40.2)	7.5(5.5-10.2)	66.0(61.4-70.3)	55.8(51.2-60.3)
55-64 years	33.6(28.1-39.4)	5.8(3.7-9.1)	50.6(43.3-57.1)	49.5(43.7-55.5)
Total pop.	24.4(22.8-26.0)	4.3(3.6-5.1)	71.3(69.4-73.2)	56.9(54.9-58.9)

The values are estimated proportions (95 % confidence interval [CI]) and are based on weighted data. Total pop = total population

### Hypertension prevalence

**Fig. 1:**
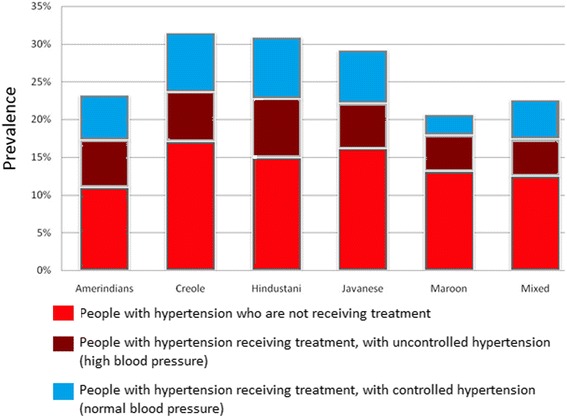
The prevalence of people with hypertension per ethnic group, presented in groups not treated or treated with uncontrolled and controlled hypertension

The estimated overall prevalence of hypertension was 26.2 % (95 % confidence interval [CI] 25.1 %-27.4 %). Fig. 1 gives the prevalence of hypertension with and without medication use in the six ethnic groups. Creole, Hindustani, and Javanese had the highest prevalence of hypertension, and Mixed and Maroons subjects had the lowest prevalence. More than 50 % of participants with hypertension were not diagnosed previously and about 25 % of those treated effectively and had normal values of blood pressure. The highest proportion of undiagnosed hypertension was found in Maroons. Hindustani and Amerindians had the largest proportion of subjects with hypertension who were treated effectively, and Maroons had the lowest.

### Ethnic differences in the prevalence of hypertension in risk factor categories

Table 4 shows that the prevalence of hypertension was higher in men among Hindustani; for Maroons and Javanese the prevalence was higher in women. The prevalence of hypertension increased with age in all ethnic groups. For demographic risk factors, among all ethnic groups except Amerindians, the prevalence of hypertension in participants living with a partner was higher compared to single participants. Residential area was only associated with the prevalence of hypertension in Maroons. The prevalence of hypertension differed according to educational level among Maroons and Hindustani. When analyzing the influence of income, the prevalence of hypertension increased for higher incomes in Creoles, whereas it decreased for lower incomes among Hindustanis. This table shows that among Hindustani the smokers, and those with physical activity less than 600 MET per week had a higher prevalence of hypertension. This corresponds with Creole subjects who smoked and Maroon subjects who performed less than 600 MET of physical activity per week. For anthropometric risk factors, we observed that the prevalence of hypertension increased with BMI for all ethnicities except for Amerindians. For all ethnic groups, the prevalence of hypertension was higher in those with a larger WC.

**Table 4:** Prevalence of hypertension in risk factor categories among six ethnic groups (*N* = 5641)

Characteristics	Amerindian	Creole	Hindustani	Javanese	Maroon	Mixed
	*N* = 427	*N* = 677	*N* = 1315	*N* = 923	*N* = 1377	*N* = 817
	Prev(CI 95 %)	Prev(CI 95 %)	Prev(CI 95 %)	Prev(CI 95 %)	Prev(CI 95 %)	Prev(CI 95 %)
Sex						
Male	27.5 % (18.8 % - 37.6 %)	28.7 % (24.2 % - 33.6 %)	33.5 % (30.4 % - 36.7 %)	25.7 % (21.7 % - 30.2 %)	17.3 % (14.0 % - 21.1 %)	23.0 % (19.2 % - 27.2 %)
Female	19.5 % (13.8 % - 26.8 %)	33.9 % (28.7 % - 39.5 %)	27.4 % (24.4 % - 30.7 %)	32.5 % (28.0 % - 37.3 %)	23.4 % (20.2 % - 27.0 %)	21.2 % (17.7 % - 25.0 %)
	*p* = 0.161	*p* = 0.143	*p* < 0.006	*p* < 0.030	*p* < 0.014	*p* < 0.509
Age group (years)						
15-24.9	9.0 % (3.8 % - 18.2 %)	9.0 % (5.2 % - 14.7 %)a	4.7 % (2.8 % - 7.6 %)a	5.2 % (2.2 % - 9.9 %)a	5.2 % (3.2 % - 8.3 %)	5.2 % (3.1 % - 8.5 %)a
25-34.9	13.0 % (4.7 % - 25.0 %)	13.4 % (8.5 % - 20.1 %)a,b	20.6 % (16.8 % - 25.0 %)a	13.9 % (8.6 % - 20.1 %)b	12.2 % (9.0 % - 16.3 %)	14.9 % (10.5 % - 20.4 %)b
35-44.9	25.5 % (14.8 % - 39.2 %)	32.9 % (24.9 % - 41.7 %)b,c	24.4 % (20.1 % - 29.2 %)b	31.6 % (25.3 % - 38.1 %)c	30.1 % (24.0 % - 37.1 %)	28.0 % (21.6 % - 34.9 %)c
45-54.9	38.8 % (24.9 % - 54.3 %)	47.6 % (39.3 % - 56.3 %)c	48.1 % (43.1 % - 53.2 %)c	44.3 % (37.1 % - 51.6 %)d	43.6 % (35.1 % - 52.8 %)	36.5 % (29.0 % - 44.9 %)c
55-64.9	43.6 % (25.4 % - 64.6 %)	67.3 % (57.0 % - 76.2 %)c	73.3 % (66.6 % - 79.3 %)d	52.6 % (42.9 % - 62.5 %)d	60.1 % (48.7 % - 70.7 %)	60.3 % (49.3 % - 70.6 %)d
	*p* < 0.001	*p* < 0.001	*p* < 0.001	*p* < 0.001	*p* < 0.001	*p* < 0.001
Living with a partner						
No	21.8 % (13.9 % - 32.3 %)	27.1 % (23.1 % - 31.5 %)	22.5 % (19.4 % - 25.9 %)	23.0 % (17.8 % - 28.8 %)	17.1 % (14.3 % - 20.3 %)	17.7 % (14.5 % - 21.3 %)
Yes	22.9 % (16.7 % - 30.0 %)	39.1 % (32.6 % - 46.0 %)	36.2 % (33.2 % - 39.2 %)	31.4 % (27.7 % - 35.4 %)	26.3 % (22.2 % - 30.8 %)	27.4 % (23.2 % - 32.1 %)
	*p* < 0.161	*p* < 0.143	*p* < 0.006	*p* < 0.05	*p* < 0.05	*p* < 0.509
Geographic area						
Urban area	22.7 % (14.2 % - 33.7 %)	30.5 % (26.8 % - 34.4 %)	29.8 % (27.5 % - 32.3 %)	28.1 % (24.6 % - 32.0 %)	16.3 % (13.4 % - 19.7 %)a	21.6 % (18.8 % - 24.6 %)
Rural coastal area	24.6 % (15.8 % - 35.6 %)	34.6 % (24.9 % - 44.7 %)	34.8 % (29.1 % - 40.7 %)	31.1 % (25.3 % - 37.3 %)	20.6 % (12.6 % - 32.0 %)a,b	24.8 % (18.0 % - 33.2 %)
Rural interior	20.5 % (12.8 % - 30.1 %)	N/A	N/A	N/A	26.5 % (22.5 % - 30.9 %)b	N/A
	*p*<0.797	*p* = 0.446	*p* = 0.113	*p* = 0.390	*p* < 0.001	*p* = 0.373
Schooling						
Primary	23.7 % (17.9 % - 30.2 %)	30.1 % (24.3 % - 36.2 %)	35.7 % (32.6 % - 39.0 %)	31.2 % (26.9 % - 35.9 %)	23.2 % (20.4 % - 26.2 %)	21.9 % (17.6 % - 27.0 %)
Secondary	21.8 % (11.3 % - 37.4 %)	34.3 % (28.0 % - 40.8 %)	27.4 % (23.4 % - 31.8 %)	28.1 % (22.8 % - 34.1 %)	11.6 % (6.7 % - 17.5 %)	21.0 % (16.8 % - 26.0 %)
Higher	2.6 % (2.1 % - 34.6 %)	30.1 % (23.5 % - 37.3 %)	18.5 % (14.2 % - 23.4 %)	25.6 % (18.0 % - 34.4 %)	11.6 % (6.2 % - 20.5 %)	20.2 % (15.5 % - 25.4 %)
	*p* = 0.253	*p* = 0.549	*p* < 0.001	*p* = 0.440	*p* < 0.001	*p* = 0.864
Working or studying						
Yes	21.8 % (15.5 % - 29.8 %)	35.8 % (27.9 % - 44.1 %)	39.7 % (35.6 % - 43.9 %)	38.1 % (31.8 % - 44.7 %)	24.6 % (20.8 % - 29.0 %)	26.5 % (20.9 % - 33.1 %)
No	23.3 % (15.8 % - 32.2 %)	29.8 % (26.0 % - 33.9 %)	26.4 % (23.8 % - 29.0 %)	25.5 % (22.0 % - 29.2 %)	18.0 % (15.2 % - 21.3 %)	20.6 % (17.8 % - 23.8 %)
	*P* = 0.816	*p* = 0.178	*p* < 0.001	*p* < 0.001	*p* < 0.008	*p* < 0.057
Income quintile in SRD						
Q1 < 800/month	21.5 % (13.3 % - 32.8 %)	18.6 % (11.5 % - 28.7 %)a	33.9 % (28.6 % - 39.6 %)	26.9 % (19.4 % - 35.9 %)	24.7 % (20.6 % - 29.4 %)	22.1 % (14.9 % - 20.2 %)
Q2 800-1499/month	32.7 % (18.0 % - 49.9 %)	27.4 % (20.4 % - 35.1 %)a,b	33.0 % (28.3 % - 38.2 %)	27.8 % (21.8 % - 34.9 %)	22.0 % (16.4 % - 28.9 %)	30.9 % (23.5 % - 38.8 %)
Q 3 1500-2199/month	21.9 % (2.5 % - 55.8 %)	40.3 % (27.9 % - 53.3 %)b,c	29.1 % (22.3 % - 36.7 %)	29.1 % (20.5 % - 39.2 %)	26.8 % (14.8 % - 40.7 %)	20.4 % (12.5 % - 30.2 %)
Q4 and Q5 > 2200/month	18.9(4.0 %-45.0 %)	42.2 % (31.2 % - 53.7 %)c	19.7 % (13.4 % - 27.3 %)	29.7 % (21.1 % - 40.2 %)	16.7 % (6.6 % - 30.0 %)	25.8 % (19.4 % - 33.2 %)
	*p* = 0.732	*p* < 0.003	*p* < 0.03	*p* = 0.965	*p* = 0.558	*p* = 0.467
Daily smoking						
No	23.1 % (17.9 % - 28.9 %)	29.0 % (25.3 % - 33.1 %)	28.8 % (26.5 % - 31.3 %)	29.5 % (26.0 % - 33.1 %)	20.8 % (18.4 % - 23.5 %)	20.0 % (17.4 % - 23.0 %)
Yes	12.1 % (1.8 % - 43.6 %)	39.1 % (30.7 % - 48.1 %)	40.0 % (34.1 % - 45.9 %)	27.3 % (20.5 % - 34.6 %)	20.6 % (11.4 % - 31.4 %)	34.0 % (26.0 % - 42.7 %)
	*p* = 0.443	*p* < 0.023	*p* < 0.001	*p*= 0.547	*p* = 0.865	*p* < 0.001
Physical activity						
< 600 MET	29.0 % (17.9 % - 41.4 %)	34.8 % (27.9 % - 42.4 %)	40.4 % (36.1 % - 44.7 %)	28.8 % (23.7 % - 34.2 %)	23.9 % (19.7 % - 28.7 %)	23.4 % (18.6 % - 28.7 %)
≥ 600 MET	18.8 % (12.2 % - 26.6 %)	29.5 % (25.1 % - 34.2 %)	26.1 % (23.4 % - 28.9 %)	28.4 % (24.3 % - 32.8 %)	18.2 % (15.1 % - 21.8 %)	21.3 % (18.0 % - 25.0 %)
	*p* = 0.75	*p* = 0.354	*p* < 0.001	*p* = 0.775	*p* < 0.05	*p* = 0.417
Body mass index						
< 23	18.4 % (9.1 % - 33.0 %)	12.4 % (8.2 % - 17.6 %)a	14.3 % (11.3 % - 18.0 %)	8.7 % (5.5 % - 13.4 %)	11.3 % (8.4 % - 15.0 %)a	9.8 % (6.5 % - 13.7 %)a
23-24.9	20.4 % (10.5 % - 35.1 %)	17.9 % (10.6 % - 26.6 %)a	29.4 % (23.1 % - 35.9 %)	22.9 % (15.3 % - 31.7 %)	9.5 % (5.2 % - 15.2 %)a	12.5 % (7.4 % - 19.0 %)a
25-27.5	17.3 % (7.0 % - 31.6 %)	41.1 % (30.4 % - 52.1 %)b	31.8 % (26.6 % - 37.4 %)	35.3 % (27.5 % - 43.8 %)a	23.3 % (16.8 % - 30.9 %)b	21.3 % (15.1 % - 29.1 %)b
27.5-30	14.0 % (5.6 % - 29.4 %)	49.7 % (38.3 % - 61.0 %)b	39.1 % (33.0 % - 45.4 %)a	36.6 % (28.1 % - 45.3 %)a,b	24.4 % (17.0 % - 32.7 %)b	29.0 % (21.1 % - 37.9 %)b
≥ 30	33.8 % (22.8 % - 45.6 %)	44.8 % (37.6 % - 52.3 %)b	44.2 % (39.4 % - 49.2 %)a	46.1 % (38.9 % - 53.3 %)b	39.4 % (33.2 % - 45.7 %)	40.1 % (33.7 % - 46.7 %)
	*p* = 0.111	*p* < 0.001	*p* < 0.001	*p* < 0.001	*p* < 0.001	*p* < 0.001
Waist circumference						
Normal	18.3 % (12.6 % - 25.8 %)	24.6 % (20.6 % - 29.0 %)	21.7 % (19.2 % - 24.3 %)	21.6 % (18.3 % - 25.2 %)	12.6 % (10.3 % - 15.3 %)	15.4 % (12.7 % - 18.5 %)
High	30.8 % (21.5 % - 40.9 %)	46.3 % (39.7 % - 53.3 %)	46.4 % (42.3 % - 50.5 %)	49.2 % (42.5 % - 56.1 %)	39.1 % (33.6 % - 44.7 %)	40.2 % (34.1 % - 46.4 %)
	*p* < 0.001	*p* < 0.001	*p* < 0.001	*p* < 0.001	*p* < 0.001	*p* = 0.033

The values are estimated proportions (with confidence interval in parentheses) and are based on data weighted for sample correction including population adjustment weights for sex and age. Pearson chi-square tests were calculated for the differences between prevalence in the subset of categories of the risk factors per ethnic group. Each subscript letter (a, b c…) denotes a subset of categories with column proportions that do not differ significantly from each other at the .05 level. N/A indicates not applicable

### Ethnic differences between the odds ratios of hypertension

Table 5 shows that in the model adjusted for age and sex only, Amerindians had a lower odds ratio (OR) for hypertension compared to Hindustani (OR: 0.7 [95 % CI 0.6-1.0]). Adding demographic factors in model 2 further reduced the odds of hypertension in Amerindians (OR: 0.5 [95 % CI 0.4-0.8]) and also showed a weaker association in Javanese (OR: 0.8 [95 % CI 0.1-1.0]). In model 3, the difference between the OR for Hindustani and Amerindians was no longer significant, although the value for the OR remained 0.7. Thus, no material changes in associations with hypertension were observed between ethnicities after additional adjustment for lifestyle and anthropometric factors (models 3 and 4).

**Table 5:** Adjusted risk of hypertension between ethnic groups

	Model 1	Model 2	Model 3	Model 4
	OR (CI)	OR (CI)	OR (CI)	OR (CI)
Hindustani	1	1	1	1
Creole	1.0(0.8-1.2)	1.0(0.7-1.3)	1.0(0.8-1.2)	1.0(0.8-1.3)
Javanese	0.9(0.7-1.1)	0.8(0.1-1.0)*	0.9(0.7-1.1)	1.0(0.8-1.2)
Mixed	0.8(0.7-1.0)	0.9(0.7-1.2)	0.8(0.6-1.0)	0.9(0.7-1.1)
Maroon	0.9(0.7-1.0)	0.9(0.6-1.2)	0.8(0.7-1.0)	0.8(0.7-1.0)
Amerindian	0.7(0.6-1)*	0.5(0.4-0.8)*	0.7(0.5-1.0)	0.7(0.5-0.9)*

*P < 0.05

Model 1 is the basic multivariate model adjusted for sex and age

Model 2 is adjusted for variables in model 1 plus demographic factors like living area, marital status, education, income, and working status

Model 3 is adjusted for variables in model 1 plus lifestyle factors like smoking and physical activity

Model 4 is adjusted for variables in model 1 plus anthropometric measures like body mass index and waist circumference

## Discussion

In this study, the prevalence of hypertension overall and in risk factor subgroups was assessed in the six major ethnic groups of Suriname. The estimated prevalence of hypertension was above 25 %. The highest prevalence was found in Creoles, Hindustani, and Javanese. Half of all hypertensive participants were not diagnosed previously and a quarter were treated effectively. We observed differences in the prevalence of hypertension between ethnic subgroups with biological, demographic, lifestyle, and anthropometric risk factors and variation in the association of ethnic groups with hypertension. The major difference in association of ethnic groups with hypertension was between Hindustani and Amerindians.

### Prevalence

The estimated prevalence of hypertension in our study is in line with previous reports from countries of the Caribbean, Latin America, and developing countries, which describe a prevalence of hypertension between 16 % and 35 % [4, 32–37]. Most studies in western countries indicate higher prevalences of hypertension in African descendants, followed by Asian descendants, Hispanics, and Caucasians [8, 12, 38–40]. In addition, studies in Amerindians report low prevalences of hypertension [41–43]. The results of our study concur with the literature for all ethnicities except for Maroons. Previous studies suggest that differences in prevalence of hypertension among adults of African descent are caused by demographic, lifestyle, and anthropometric factors [44–46]. This is supported by our results, as the ethnic groups with the highest prevalence of hypertension (Creoles) and the lowest prevalence (Maroons), are both from African descent and have similar biological characteristics.

### Awareness and treatment

In this study, awareness in hypertensive participants falls within the range of other developing countries, but the proportion of adequately treated subjects is higher. In other developing countries, it has been estimated that between 23 % and 71 % of all hypertensive people were aware of their condition and 4 % to 16 % were adequately treated [4, 47]. Success rates may be high in Suriname compared to other developing countries [4, 48], because of the country’s extensive primary health care system [49]. However, awareness and the ratio of effectively treated patients is higher in developed countries. In the United States, just over 80 % of hypertensive subjects were aware of their condition and slightly more than 50 % were treated effectively [50]. Besides dissimilar resources between developed and developing countries, there are also differences in ethnic composition. Thus, in addition to the differences in compliance with treatment and availability of medication, different responses to antihypertensive treatment by ethnic groups should also be considered to explain discrepancies in the efficacy of treatment [20]. This argument is supported by Amerindians having the highest levels of effective treatment and Maroon subjects having the lowest. Therefore, ethnic-specific treatment for hypertension may be indicated and should be explored further. The findings of this study indicate that more effort must be taken to improve awareness by people with hypertension and efficacy of treatment.

### Risk factors

As in previous studies, the results in our study for sex-specific prevalence are ambiguous [51, 52]. We observed a higher prevalence of hypertension for men among Hindustani subjects and for women in Maroons and Javanese. The findings of our study in regards to age are also in line with the literature, as hypertension increases with age regardless of race and ethnicity [53]. However, the age- and sex-adjusted risk for hypertension was equal for all ethnicities, with exception of the Amerindians for whom the risk was lower. Biological mechanisms that explain higher blood pressure levels in people of African descent are described in the literature, but to our knowledge, mechanisms explaining lower blood pressure levels in Amerindians are not known and need to be studied in more depth. Neither age nor sex are modifiable risk factors. However, they may be relevant for identification of groups at risk.

Demographic factors like education, income, employment, and area of residence have been associated with hypertension [54–60]. In this study, the prevalence of hypertension increased with income in Creoles, living in the rural interior in Maroons, and living with a partner in Hindustani, Javanese, and Maroons. Prevalence decreased with education in Hindustani and Maroons, with employment in Hindustani, Javanese, and Maroons, and with level of income in the Hindustani. After adjusting for all risk factors, we measured a weaker association of Javanese and Amerindians with hypertension compared to Hindustani, which might indicate that some of these factors influence the level of hypertension for these two ethnic groups. Previous studies report higher prevalences of hypertension in urban areas compared to rural areas [61–63]. Published results on physical activity from the Steps Survey 2013 in Suriname, report the percentage of required physical activity in the rural coastal area compared to the urban area [64]. A lower percentage of required physical activity could be associated with the higher prevalence of hypertension measured in the rural coastal area in our study. Possibly, our observations in this study of Maroons having higher levels of employment, higher levels of education, and lower prevalences of hypertension, are related to the fact that they are more likely to live in urban areas. In such a case, it is plausible that these factors have an impact on the prevalence of hypertension in the urban setting. However, no previous studies have compared hypertension in urban and rural settings in Suriname, and the higher prevalence observed in Maroons living in rural coastal and rural interior areas requires further research.

The results clearly demonstrate that the prevalence of hypertension in demographic risk factor subgroups differed between ethnic groups. We also observed a change in the different associations of ethnic groups with hypertension after adjusting for demographic factors. These results imply that achievement of uniform intervention programs will be limited and tailor-made programs should be developed.

For lifestyle factors, the prevalence of hypertension was higher in Hindustani and Creole smokers, and in Maroon and Hindustani subjects performing less than 600 MET of physical activity per week. Previous studies show that lifestyle factors like low physical activity and cigarette smoking are associated with hypertension [65–68]. These risk factors are modifiable and are valuable points of interest for intervention programs.

Many studies show that high BMI or WC are risk factors of hypertension [69–71]. In this study, the prevalence of hypertension increased with BMI and WC categories for all ethnicities. The BMI subgroups for Amerindians, however, were too small to show significant differences at a 0.05 level, thus the increase with BMI categories for this group could not be tested for statistical significance. Adjustment for these anthropometric factors in addition to sex and age did not affect the association with hypertension, and the OR remained lower in Amerindians. The results suggest that public health interventions focusing on both BMI and WC control should not be aimed at specific ethnic groups; however, in order to confirm this suggestion, this association should be explored in additional studies.

The major difference in association of ethnic groups with hypertension was between Hindustani and Amerindians. Differences in associations of ethnic groups with hypertension were not materially affected by adjustment for lifestyle factors or anthropometric factors. The differences in associations of the ethnic groups with hypertension were mostly influenced by demographic risk factors, and these should be addressed accordingly.

### Strength and limitations

Strengths of this cross-sectional study include the design, with a stratified multistage cluster and a large sample size, which was adequate to represent the ethnic and geographic diversity of the Surinamese population by sex in five different age groups [24]. The design included measures like the Kish method and standardized data collection tools to minimize selection and interviewer bias [24, 27]. In addition, in the analysis, sample weights were applied to correct for selection bias. Further, the percentages of missing data in general were relatively small (1.1 %), except for income status (30.2 %). However, the model that included income as a confounder fit and was not compromised (goodness to fit test). Still, some limitations should be considered regarding this study. First, despite the overall large sample size of the study some risk factors were present in only a few participants when analyzed per ethnic subgroup. In these cases, the sample size was too small to measure statistical differences between the observed hypertension rates at a significance level of 0.05. Second, although the wide range of variables evaluated in this study allowed us to control for confounders, residual confounding might still have occurred, as with any observational study. For example, information on nutrition was not considered in this paper.

## Conclusion

The results of the present study showed that the prevalence of hypertension in Suriname was in the range of developing countries. By ethnic group, the highest prevalence was found in Creoles and the lowest in Maroons. Next to Creoles, Hindustani and Javanese also had high prevalences of hypertension, and Amerindians exhibited a low prevalence, after Maroons. In the analysis, Amerindians had the lowest OR for hypertension, in comparison to Hindustani. The differences observed in the prevalence of hypertension for risk factor subgroups between and within ethnic groups allow us to generate valuable ethnic-specific hypotheses, which are important for research on the development of tailor-made public health interventions.

## Abbreviations

BMI: Body mass index
GPAQ: Global Physical Activity Questionnaire
MET: Metabolic Equivalent of Task
NCD: Non-communicable disease
PAHO: Pan American Health Organization
WC: Waist circumference
WHO: World Health Organization
